# Estimating utility values for non-alcoholic steatohepatitis health states: a discrete choice experiment

**DOI:** 10.57264/cer-2023-0033

**Published:** 2024-01-16

**Authors:** Daniel Aggio, Katy Gallop, Villum Wittrup-Jensen, Soulmaz Fazeli Farsani, Andrew J Lloyd

**Affiliations:** 1 Acaster Lloyd Consulting Ltd, London, WC1X 8NL, UK; 2 Boehringer Ingelheim International GmbH, D-55216 Ingelheim/Rhein, Germany

**Keywords:** discrete choice experiment, EQ-5D, health-related quality of life, NASH, non-alcoholic steatohepatitis, qualitative, utilities

## Abstract

**Background:**

This study estimated utility values for non-alcoholic steatohepatitis (NASH). Previous studies have assumed that health-related quality of life does not vary between the early stages of NASH.

**Materials & Methods:**

Discrete choice experiment (DCE) surveys estimated the value of avoiding fibrosis progression. Patients also completed the EQ-5D-5L. Marginal rates of substitution estimated utility change associated with fibrosis progression.

**Results:**

DCE surveys were completed by the UK general public (n = 520) and patients with NASH (n = 154). The utility decline between fibrosis stages F1 and F4 decompensated was between -0.521 to -0.646 (depending on method).

**Conclusion:**

Three methods were used to estimate utilities for NASH, each one showed sensitivity to advancing fibrosis, including in the early stages, which is often considered asymptomatic.

## Plain language summary

**What is this article about?:**

Nonalcoholic steatohepatitis (NASH) is a form of nonalcoholic fatty liver disease (NAFLD). In addition to excessive fat in the liver, NASH also includes liver inflammation and damage. Over time, NASH can cause permanent liver damage. As the condition worsens, it can have a more severe impact on patients' health-related quality of life. However, few studies have explored the impact of NASH on patients at different stages of the condition, particularly in the early stages. This study explored the impact of NASH on health-related quality of life at different disease stages.

**What were the results?:**

Patients with NASH experience significant impacts on health-related quality of life, which becomes progressively worse as the condition worsens, even in the early stages of disease.

**What do the results of the study mean?:**

The results show the importance patients with NASH and the public place on avoiding worsening NASH and the potential benefits of preventing worsening NASH. These results could also be used to estimate the cost-effectiveness of new NASH treatments.

## Tweetable abstract

Preventing worsening in liver fibrosis can produce net gains in health and quality of life. This new research explores methods for valuing these gains in health

Nonalcoholic steatohepatitis (NASH) is the progressive form of nonalcoholic fatty liver disease (NAFLD). NASH is the inflammatory subtype of NAFLD and is characterized by steatosis as well as evidence of hepatocyte injury (ballooning) and inflammation, with or without fibrosis [1]. Indeed, NASH is classified in five stages according to levels of fibrosis ranging from none (stage F0) to cirrhosis (F4), which can lead to end-stage liver disease and hepatocellular carcinoma (HCC) [1–3]. Comorbidities such as obesity, high blood pressure and diabetes increase the risk of NAFLD progressing to NASH [4]. One study estimated the overall global prevalence of NASH to be 1.5–6.5% [5]. Currently, there are no approved treatments for NASH, but there are a number in late stages of clinical development. The present standard of care is lifestyle modification, although some patients are prescribed off-label pharmacologic treatments, such as vitamin E, statins and metformin [6].

NASH has often been described as an asymptomatic condition [7] particularly in its early stages, however studies are increasingly recognizing that individuals with NASH do experience symptoms such as fatigue, malaise, pruritis and abdominal pain [6,8]. A recent review showed that people living with NASH had lower health-related quality of life (HRQL) compared with the general population and people with NAFLD [9]. This review described the different impacts of NASH on patients' daily life, including dietary restrictions, reduced sleep, negative impacts on relationships, limited social activity, medication/healthcare related frustration and fear or worry about the future. There is also emerging evidence to suggest that worsening fibrosis has a more severe impact on HRQL [8–10].

The adoption of new treatments in many countries requires evidence of the HRQL impact of the condition and treatment. For agencies such as the National Institute for Health and Care Excellence (NICE) in the UK, the HRQL data should be measured as utility weights which are used to estimate quality adjusted life years (QALYs). Utility weights reflect the perceived value of health on a scale from 0 (equal to dead) to 1 (full health) and should reflect societal preferences. Previous research in NASH has presented utility scores for early fibrosis stages grouped together, suggesting an assumption that there is no difference in HRQL between these early stages (e.g., O'Hara *et al.*, reported utilities for patients at F0, F1 and F2 to be 0.80) [11]. Similarly, Zhang *et al.*, reported a cost-utility analysis including a utility of 0.84 for patients in F1, F2 and F3 [12]. It may be correct that HRQL does not vary in the early stages of NASH, but this is an empirical question and should not be based on an assumption.

Probably the most common method for generating utility weights is the EQ-5D [13]. However, the EQ-5D is often criticized for being insensitive to changes in some conditions, and in such circumstances alternative methods can be used. In this study we also explore the use of discrete choice experiment (DCE) methods for estimating utilities for different fibrosis stages. DCEs are an alternative method for estimating utilities where the survey includes attributes which describe quality of life and length of life [14]. The objectives of this study were to explore the HRQL impact at each stage of NASH and to then use different methods to value differences in HRQL associated with NASH fibrosis progression. The study also aimed to explore the sensitivity of the EQ-5D-5L to changes in fibrosis stage.

## Methods

### Study design

This study used a two-phase cross-sectional design consisting of qualitative interviews and online surveys. Both phases were reviewed by the Western Institutional Review Board (WIRB) and determined to be minimal risk and exempt from full review. All participants gave informed consent.

#### Qualitative phase

To explore the HRQL burden at each fibrosis stage, semi-structured interviews were conducted with NASH patients from the UK and USA with either biopsy or fibroscan confirmed fibrosis stage. Participants were recruited by a specialist patient recruitment agency. Interviews followed a semi-structured interview guide and explored symptoms and HRQL impacts. Participants were probed regarding the severity and frequency of any symptoms they mentioned. Questions also explored daily activities, emotional impact, work relationships, self-care,= and leisure activities. Interviews lasted 60 to 80 min and were recorded and transcribed.

Qualitative data were analysed using thematic analysis in MAXQDA [15]. This involved reading and re-reading the transcripts; generating a coding framework and then coding the transcripts. This then led to the identification of themes and relationships between themes.

#### Utility survey

The online survey was designed to estimate utility decrements associated with fibrosis stage progression. Separate surveys were developed for patients and the general public to derive patient and societal utilities, respectively. The development of the DCE was informed by the qualitative results and ISPOR best practice guidelines [16,17]. The DCE section of the survey started with an introduction to the task and, for the public version, background information about the condition (as shown in Appendix 1); the general public were asked to imagine they had a liver disease and were at stage F2. Patients with NASH were asked to respond to the survey questions in the context of their own fibrosis stage.

The DCE surveys described NASH outcomes in terms of different attributes which were combined into hypothetical choices. The survey also contained sociodemographic questions (including age, gender, ethnicity and employment status). Additional clinical questions were included in the patient survey to capture information about diagnosis, comorbidities and current fibrosis stage.

### Survey development

#### Identification of survey attributes

A review of published studies, clinical trials, and regulatory documents (such as the Summary of Product Characteristics) and other grey literature was conducted. Attribute selection was also driven by the findings from the qualitative interviews with NASH patients [9,18–21].

A long list of possible attributes was identified for inclusion in the online DCE survey. The selection process was framed by the study objectives, and attributes were selected which were considered important from a patient perspective, and avoided potential overlap with other attributes. Attributes describing length of life and fibrosis stage attributes were included to estimate utility weights. As the symptoms and impacts of NASH correlate with fibrosis stage, it was decided to incorporate these concepts into the description of fibrosis stage. The DCE survey included length of life, fibrosis stage and mode of treatment administration. Draft descriptions of the attributes and attribute levels were developed based on the literature and the qualitative findings.

#### Survey content

Surveys were developed for patients and the general public. For the public version, participants were asked to imagine they were in F2 and the attribute levels described an improvement (by 1 stage), remain the same (F2), worsen by 1 stage (F3), worsen by 2 stages (F4 compensated), worsen by 3 stages (F4 decompensated). Length of life was also described in terms of five levels (overall length of life reduced by 12, 9, 6 or 3 years or no reduction). In the patient version, participants were asked to consider their own current fibrosis stage when considering the choice scenarios. This attribute was described according to two levels (fibrosis worsens by 1 stage, fibrosis remains the same). Length of life was described based on three levels (reduced by 12, 6 or 0 years). Both versions of the survey described treatment mode according to the same three levels (daily tablet, injection once a month and injection biweekly). The attributes and levels were combined into choice sets using a D-efficient design generated with NGene software version 1.2.1 [22]. The survey consisted of 15 choice sets for the general public version and 12 choice sets for the patient versions. Sample choice questions are shown in Appendix 2. The survey included background questions, and the patient survey included the EQ-5D-5L [13] and the NASH-Check (a disease-specific measure of HRQL) [23].

The surveys were reviewed in cognitive debrief interviews with NASH patients (n = 5), the general public (n = 5) and clinicians with experience of treating people with NASH (n = 2). The aim of these interviews was to check the clinical accuracy of the survey, patient relevance and understanding among patients and the public. Participants were asked to speak aloud as they read/completed the survey and provide feedback. Minor amendments were made after all interviews were completed based on the interview findings. The final attributes and levels for each survey are shown in Table 1.

**Table 1.** General public and patient DCE survey attributes and levels.

Attribute	Level 1	Level 2	Level 3	Level 4	Level 5
General Public DCE					
Length of life	Overall life expectancy reduced by 0 years	Overall life expectancy reduced by 3 years	Overall life expectancy reduced by 6 years	Overall life expectancy reduced by 9 years	Overall life expectancy reduced by 12 years
Disease (fibrosis) stage	Stage 1	Remain stable/Stage 2	Stage 3	Stage 4	Stage 5
How treatment is given	Treatment is a tablet taken daily at home	Treatment is an injection once a month	Treatment is an injection every 2 weeks		

Patient DCE					
Length of life	Overall life expectancy reduced by 0 years	Overall life expectancy reduced by 6 years	Overall life expectancy reduced by 12 years		
Disease (fibrosis) stage	Worsen by 1 stage over next 4 years	Remain stable over next 4 years			
How treatment is given	Treatment is a tablet taken daily at home	Treatment is an injection once a month	Treatment is an injection every 2 weeks		

DCE: Discrete choice experiment.

#### Pilot

The DCE survey was piloted with the UK general public (n = 71, results not shown) to explore whether attribute coefficients were in the expected direction and to establish quality control thresholds for the survey data (e.g., excluding members of the general public completing the survey too quickly). A few minor amendments to the wording of the attribute descriptions were made to improve clarity. Based on an analysis of pilot data, participants who completed the DCE survey in <230 seconds were excluded from the main study analyses (estimated based on a reasonable minimal completion time and an optimal threshold for excluding respondents making ≥1 illogical responses).

#### Main study participants

Participants for the general population survey were recruited in the UK between March and April 2021 with sample quotas for age, gender, ethnicity and geographic location. Patients with NASH were recruited by a specialist patient recruitment agency. They were all aged over 18, had a diagnosis of NASH with their fibrosis stage confirmed by biopsy or fibroscan (participants did not specify which procedure was used for staging), had a fibrosis stage between 1 and 4 (compensated or decompensated) or HCC and lived in the UK or US. No formal sample size estimation was conducted; target sample sizes for each sample were based on sample sizes reported in previous valuation studies [24,25], best practice guidance [16,26] and recruitment feasibility. All eligible participants provided online informed consent.

#### Statistical analyses

Sociodemographic and clinical data were analyzed using descriptive statistics. NASH-CHECK data were scored based on the developers' scoring guidelines. EQ-5D-5L was scored with UK and US preference weights [27,28]. Discrete choice data were analyzed using a mixed-effects logit regression model [29] to estimate preference strength for each attribute while accounting for heterogeneity among respondents. These models are estimated using the maximum simulated likelihood approach. The logit model estimates beta coefficients for each attribute. For categorical variables, the coefficient indicates their importance with respect to a reference category. For the continuous reduction in life expectancy variable, the model presents the odds of selection for a 1-year reduction in life expectancy. Coefficients were converted to odds ratios (ORs) for ease of interpretation. An alternative-specific constant was added to the model to account for any ‘left’ bias (i.e., selecting the choice shown on the left more frequently) in respondent's choices.

The utility value for a change in fibrosis stage was estimated based on the marginal rates of substitution (MRS), which is estimated by taking a ratio of the coefficients for two attributes: the life expectancy coefficient for a 1-year reduction (obtained by entering life expectancy as a continuous variable into the models) and the change in fibrosis stage attribute. The MRS indicates the extent to which participants were willing to trade years of life to avoid certain levels of worsening fibrosis. The change in utility was calculated by dividing the MRS value for each fibrosis attribute level by the estimated remaining life expectancy of the sample. The life expectancy of the sample was estimated based on the average life expectancy for men and women in the UK and weighted according to the proportion of men and women in the sample [30]. Average remaining life expectancy for the patient and general public samples was 30.2 years and 34.4 years, respectively. In order to enable comparison of the utilities estimated using the different methods, the utilities for each NASH stage were then estimated using utility values for a change in NASH stage derived from the general public DCE, anchored using the patient EQ-5D estimates for NASH stage F2 based on UK preference weights, as shown in the equation below.

NASH stage utility value x = patient EQ-5D F2 utility value + utility for y stage improvement/worsening

Where x represents the NASH stage; y represents the general public DCE estimated change in utility for a specified change in NASH stages.

All quantitative data were analyzed in Stata version 16.0.

#### Supplementary analysis: predictors of EQ-5D utility values

An additional analysis was performed to explore the predictors of EQ-5D utility values, based on the UK preference weights, and to understand whether current fibrosis stage predicts EQ-5D utility values independent of the comorbidities that often accompany NASH. Linear regression models were used to identify univariate predictors of EQ-5D values. Significant univariate predictors were then included in a multivariate model to identify independent associations.

## Results

### Qualitative interviews

Semi-structured interviews were conducted with 17 NASH patients (with biopsy or fibroscan confirmed fibrosis stage) from the US or UK. Patients at all fibrosis stages reported symptoms they experience due to NASH. Fatigue (n = 17) was common at all stages and was more severe at advanced stages. The severity of the fatigue described ranged from feelings of tiredness through to chronic exhaustion.

*“It's almost like you just feel whacked, like just not having any more energy to do anything. (NASH patient, F3)*

Abdominal pain (n = 16) was also common at all stages, with some participants describing a stabbing/throbbing pain and others an aching/dull pain. Participants reported more frequent or more severe pain at more advanced fibrosis stages.

*“Some days it would feel like somebody was stabbing me” (NASH patient, F1)*

*“It's a real digging under the ribs, a real sharp pain.” (NASH patient, F2)*

Other common symptoms included cognitive problems (n = 14), sleep issues (n = 15), itchy skin (n = 11) and nausea/vomiting/bloating (n = 10) but the impact of fibrosis stage on severity of these symptoms was less clear. Sweating (n = 3), bowel issues (n = 3) and weight gain (n = 3) were also reported but were less common. In addition to the symptomatic burden, patients also described how the symptomatic burden negatively impacted many areas of daily life and wellbeing, including daily, social and physical activities, emotional well-being, relationships and work.

*“I mean going out with friends for one is not something that I do, because most days I feel sluggish and tired and in pain” (NASH patient, F2)*

Impacts were more severe at advanced stages (F3 and F4), except for social activities and diet which had a similar impact across stages. Further detail on the results of the qualitative phase of this study have been published elsewhere [21].

### General public survey sample

The general public sample (n = 520) were largely representative based on the UK census data but were slightly older than the population norm (Table 2) [31]. The prevalence of long-term conditions was also slightly higher than that observed in the latest UK opinions and lifestyle survey [32].

**Table 2.** Descriptive statistics of General Public (n = 520)^†^ and NASH patient samples (n = 154).

		General public (n = 520)	NASH patient sample (n = 154)	UK census data
Characteristic		% (n)		
Age (years)	Mean (SD)	48.5 (15.9)	52.7 (11.7)	39.4^‡^
	Range	18–88	24–72	
Country	UK	100% (520)	10.4% (16)	
	USA	NA	89.6% (138)	
Gender	Male	48.1% (250)^§^	43.5% (67)^¶^	49%^‡^
Ethnicity	White British or American	91.5% (476)^#^	42.2% (65)^††^	86%^‡^
Employment status	Employed full or part-time	58.6% (305)^‡‡^	33.1% (51)^§§^	62%^‡^
Long-term condition	Yes	44.6% (232)	NA	36%^¶¶^
	No	54.8% (285)	NA	
	Prefer not to answer	0.6% (3)	NA	
Time since NASH diagnosis (months)	Mean (SD)	NA	63.0 (57.4)	
	Range	NA	2–376	
	Median (IQR)	NA	43.5 (22.5–88.0)	
	Missing	NA	1.3% (2)	
Time since last biopsy/fibroscan (months)	Mean (SD)	NA	14.1 (33.9)	
	Range	NA	0–376	
	Median (IQR)	NA	8.0 (4.0–16.0)	
	Not known	NA	2.6% (4)	
Current fibrosis stage	Stage 1	NA	26.0% (40)	
	Stage 2	NA	24.0% (37)	
	Stage 3	NA	27.9% (43)	
	Stage 4 compensated cirrhosis	NA	12.3% (19)	
	Stage 4 decompensated cirrhosis	NA	9.7% (15)	
	Liver cancer/hepatocellular carcinoma	NA	0.0% (0)	
Chronic conditions	Cardiovascular disease	2.9% (15)	12.3% (19)	
	Type 2 diabetes	7.3% (38)	33.3% (51)	
	Type 1 diabetes	1.4% (7)	0.7% (1)	
	High blood pressure	17.1% (89)	35.1% (54)	
	High cholesterol	12.5% (65)	32.5% (50)	
	Obesity	8.5% (44)	18.2% (28)	
	Depression	13.1% (68)	18.2% (28)	
	Anxiety	15.0% (78)	13.0% (20)	
	Other	13.9% (72)	9.7% (15)	
NASH symptoms/complications^##^	Ascites	NA	20.1% (31)	
	Gastrointestinal bleeding	NA	9.1% (14)	
	Sleep apnoea	NA	29.2% (45)	
	None of above	NA	46.1% (71)	

† all self-reported.

‡ Figures based on data from the 2011 United Kingdom national census for England and Wales (Office for National Statistics https://www.ons.gov.uk/census).

§ Other response options (Female: 51.7% [n = 269], Other: 0.0% [n = 0], Prefer not answer: 0.2% [n = 1]).

¶ Other response options (Female: 56.5% [n = 87], Other: 0.0% [n = 0], Prefer not answer: 0.0% [n = 0]).

# Other response options (Black: 1.4% [n = 7], Asian: 4.8% [n = 25], Mixed: 1.5% [n = 8], Other: 0.2% [n = 1], Prefer not to answer: 0.6% [n = 3]).

†† Other response options (Black American: 22.7% [n = 35], Hispanic and Latino Americans: 13.6% [n = 21], Asian British/American: 8.4% [n = 13], Mixed British/American: 5.8% [n = 9], Other: 2.6% [n = 4], Prefer not to answer: 4.5% [n = 7]).

‡‡ Other response options (Full-time homemaker/caregiver: 6.2% [n = 32], Retired: 21.7% [n = 113], Seeking work/unemployed: 5.8% [n = 30], Unable to work due to disability: 4.0% [n = 21], Student: 2.7% [n = 14], Other: 0.8% [n = 4], Prefer not to answer: 0.2% [n = 1]).

§§ Other response options (Full-time homemaker/caregiver: 11.0% [n = 17], Retired: 25.3% [n = 39], Seeking work/unemployed: 4.6% [n = 7], Unable to work due to disability: 18.8% [n = 29], Student: 0.0% [n = 0], Other: 6.5% [n = 10], Prefer not to answer: 0.7% [n = 1]).

¶¶ Figure based on data from the 2013 UK Opinions and Lifestyle Survey (Office for National Statistics https://www.ons.gov.uk/peoplepopulationandcommunity/healthandsocialcare/healthandlifeexpectancies/compendium/opinionsandlifestylesurvey/2015-03-19/adulthealthingreatbritain2013.

## All self-reported, therefore may not be clinically diagnosed or confirmed.

NASH: Non-alcoholic steatohepatitis; SD: Standard deviation.

### Patient survey sample

The patient sample (n = 154) are shown in Table 2. All fibrosis stages from F1 to F4 decompensated were captured, with most patients currently at F1 to F3 (77.9%). A wide range of comorbidities were reported, with most patients reporting at least one comorbidity (80.5%).

### General public DCE survey results

All attributes were significant (p < 0.001) independent predictors of choice (Table 3). Participants preferred to avoid a reduction in years of life. The odds of preferring an outcome which reduced length of life by one year was 0.675, (95% confidence intervals, 95% CI = 0.646 to 0.705). By extrapolating the coefficient for one year, the estimated odds of preferring an outcome which reduced length of life by twelve years was close to zero (OR: 0.009, 95% CI = 0.005, 0.015). Participants also placed significant weight on avoiding worsening fibrosis. The odds ratio was lowest for scenarios associated with a 3-stage worsening (OR: 0.003, 95% CI: 0.002 to 0.006). Treatments that were administered via an injection were more likely to be chosen compared with oral tablets; the highest odds ratio was observed for biweekly injections (OR: 3.514, 95% CI: 2.716 to 4.545).

**Table 3.** Results of the mixed-effects logit regression model of participant choices for life expectancy, fibrosis stage and mode of treatment administration among the UK general public (n = 520).

	Coeff.	OR	95% CI	p-value
Alternative-specific constant Ref.: treatment B (right column)	0.107	1.113	(1.006, 1.230)	0.038
Reduction in life expectancy 1-year decrease	-0.393	0.675	(0.646, 0.705)	<0.001
Fibrosis stage				
Improved by 1 stage (stage F1)	1.373	3.947	(2.914, 5.347)	<0.001
Remained the same (stage F2)	Reference			
Worsened by 1 stage (stage F3)	-0.889	0.411	(0.337, 0.502)	<0.001
Worsened by 2 stages (stage F4 compensated)	-2.311	0.099	(0.073, 0.134)	<0.001
Worsened by 3 stages (stage F4 decompensated)	-5.681	0.003	(0.002, 0.006)	<0.001
How treatment is given				
Tablet (once daily)	Reference			
Injection (monthly)	0.402	1.495	(1.305, 1.714)	<0.001
Injection (biweekly)	1.257	3.514	(2.716, 4.545)	<0.001

F1: Fibrosis stage 1; F2: Fibrosis stage 2; F3: Fibrosis stage 3; F4: Fibrosis stage 4; CI: Confidence interval; OR: Odds ratio.

#### Estimation of utility for change in fibrosis

Based on the MRS estimates rescaled against remaining average life expectancy, utility values for an increase and decline in utility (disutility) were estimated (Table 4). Larger disutilities were observed for worsening to more advanced fibrosis stages, with a particularly marked decline observed for worsening to stage F4 decompensated compared with remaining at stage F2 (-0.420, 95% CI: -0.372, -0.467).

**Table 4.** Estimated utilities and disutilities for different fibrosis stages based on patient EQ-5D data and general public DCE results.

	Patient survey	General public DCE (n = 520)	
Fibrosis stage	EQ-5D UK utilities (95% CI), n	Change in utility (95% CI)^†^	Estimated utility (95% CI)^‡^
F1	0.768 (0.689, 0.848), 40	+0.101 (0.079, 0.124)	0.772 (0.750, 0.795)
F2	0.671 (0.607, 0.736), 37	Ref.	-
F3	0.513 (0.439, 0.587), 43	-0.066 (-0.051, -0.080)	0.605 (0.591, 0.620)
F4 compensated	0.252 (0.097, 0.407), 19	-0.171 (-0.148, -0.193)	0.500 (0.478, 0.523)
F4 decompensated	0.122 (-0.041, 0.285), 15	-0.420 (-0.372, -0.467)	0.251 (0.204, 0.299)

† Positive values indicate an increase in utility, whereas negative values represent a utility decline (or a disutility).

‡ Estimated using from the change in utility values derived from the general public DCE anchored using the patient EQ-5D estimates for F2 based on UK preference weights (0.671) (e.g., F1 = 0.671 + 0.101 = 0.772).

F1: Fibrosis stage 1; F2: Fibrosis stage 2; F3: Fibrosis stage 3; F4: Fibrosis stage 4.

### Patient sample DCE survey results

All attributes were significant (p < 0.001) independent predictors of choice (Table 5). People with NASH expressed a preference to avoid a reduction in years of life (per year of life lost: OR 0.611, 95% CI: 0.528, 0.709) and worsening fibrosis (per 1-stage worsening: OR 0.010, 95% CI: 0.003, 0.036). By extrapolating the coefficient for one year of life lost, the estimated odds of preferring an outcome which reduced length of life by twelve years was close to zero (OR: 0.003, 95% CI = 0.000, 0.016). Patients were also averse to receiving treatment via injections; monthly (OR: 0.365, 95% CI: 0.213, 0.624) and biweekly injections (OR: 0.072, 95% CI: 0.030, 0.171) were significantly less likely to be chosen compared with daily tablets.

**Table 5.** Results of the mixed-effects logit regression model of patient preferences for life expectancy, fibrosis stage and mode of treatment administration (n = 154).

	Coeff.	OR	95% CI	p-value
Alternative-specific constant Ref.: treatment B (right column)	-0.231	0.794	(0.591, 1.065)	0.124
Reduction in life expectancy 1-year decrease	-0.492	0.611	(0.528, 0.709)	<0.001
Fibrosis stage				
Remained the same	Reference			
Worsened by 1 stage	-4.655	0.010	(0.003, 0.036)	<0.001
How treatment is given				
Tablet (once daily)	Reference			
Injection (monthly)	-1.008	0.365	(0.213, 0.624)	<0.001
Injection (biweekly)	-2.634	0.072	(0.030, 0.171)	<0.001

F1: Fibrosis stage 1; F2: Fibrosis stage 2; F3: Fibrosis stage 3; F4: Fibrosis stage 4; CI: Confidence interval; OR: Odds ratio.

#### Estimation of utility for change in fibrosis

Based on the mixed effects logit model results for the whole patient sample, the disutility for a 1-stage worsening in fibrosis was -0.313 (95% CI: -0.223, 0.-403). Further analyses were conducted stratifying the sample by fibrosis stage (stage 1–2; stage 3; stage 4 compensated and decompensated). Due to small subgroup sample sizes, these analyses were conducted using conditional logit models, which do not account for preference heterogeneity across the sample. Using these results, larger disutilities for a 1-stage worsening in fibrosis were estimated for patients at more advanced fibrosis stages (data provided in Appendix 3).

**Figure 1.**
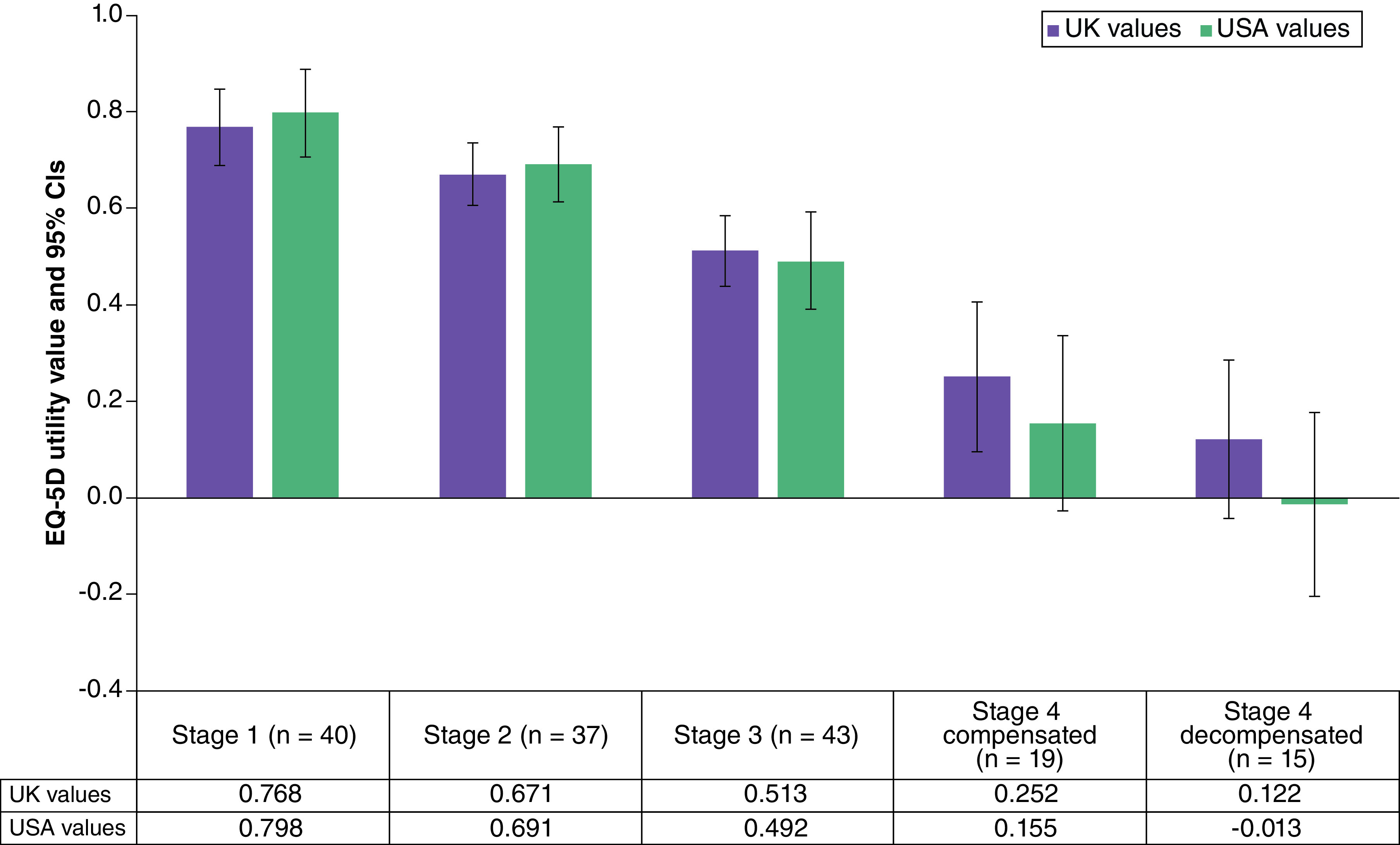
EQ-5D utility values by fibrosis stage based on UK and US preference weights (n = 154). CI: Confidence interval.

Patient utilities were also estimated for each fibrosis stage using patient reported EQ-5D scores. Utilities were estimated for all patients using both UK and USA preference weights. Utility values were lower for more advanced fibrosis stages (Figure 1). Utilities declined between each fibrosis stage, but the largest decrement was reported between stage 3 and stage 4 compensated. US and UK utility values were comparable at early fibrosis stages but US values were markedly lower than UK values at more severe stages. A comparison between patient-derived utilities based on EQ-5D values, estimated using UK preference weights, and utilities derived from the general public DCE, anchored using the patient EQ-5D F2 utility value, is shown in Table 4. The utility values are comparable for fibrosis stage 1, but the general public estimates are substantially higher for fibrosis stages ≥3 when compared with the patient-derived data.

#### NASH check results

People in more advanced fibrosis stage reported worse symptom scores on the NASH-CHECK. Large differences between F1 and F4 fibrosis stage were observed for the abdominal pain, abdominal bloating and physical fatigue scores. Large differences were also observed across all subscales, but they were particularly marked for social impact and activity limitations. All NASH Check results are provided in the Appendix 4.

#### Supplementary analysis: predictors of EQ-5D utility values

Significant univariate predictors (p < 0.05) of EQ-5D utility values included current fibrosis stage, age, years diagnosed with NASH, employment status, cardiovascular disease, Type 2 diabetes, high cholesterol, obesity, depression and anxiety. Multivariate analysis showed that only current fibrosis stage, cardiovascular disease, depression and anxiety were significantly associated with EQ-5D utility values after adjusting for all other significant univariate predictors (see Appendix File 5). This suggests that current fibrosis stage is associated with EQ-5D utilities independently of other comorbidities. Years diagnosed with NASH and employment were not included in the multivariate model as they were moderate to highly correlated with age (r = 0.39–0.59) and the variance inflation factor indicated some evidence of multicollinearity (values for abovementioned variables ranged between 2.35 and 3.87).

## Discussion

This study was designed to explore if HRQL is different in different NASH fibrosis stages. Qualitative methods, HRQL assessments and stated preference surveys were used to explore this issue and the study produced quite consistent results. Patients in more severe NASH fibrosis stages reported more symptoms and a greater burden of disease in the qualitative research undertaken. In the quantitative phase, we also found that patients in more severe fibrosis stages reported worse HRQL using the EQ-5D-5L and the disease-specific NASH-CHECK measure. People with NASH had a strong preference to avoid worse liver fibrosis. In a parallel preference survey, the general public also expressed a strong preference to avoid worse fibrosis stages which was used to provide an alternative estimate of health state utility. Also, there was no evidence from the current study that EQ-5D was not sensitive enough to measure the effect of fibrosis on HRQL. The study provides evidence from different sources that HRQL burden increases with worsening liver fibrosis.

We believe that this study advances our understanding of the impact of the early stages of NASH on patients. Previous studies reporting utilities for NASH have assumed that HRQL is the same between F0 to F3 [11,12], however as the current study found a utility difference of 0.255 between F1 and F3 (from the patient EQ-5D data) this assumption was not supported by the current study. Making this assumption in a cost–effectiveness model may miss important aspects of benefit in treatments that are effective in delaying or preventing further decline in fibrosis. There may often be merit in testing assumptions regarding a model structure with patient-derived data. We believe that this study provides an example of why incorporating patient views in cost–effectiveness model structures can be a valuable step. The qualitative data illustrated the nature of the patient burden experienced. The subsequent survey data was able to quantify that. Declines in HRQL may be explained by a wide range of impacts, including worsening symptoms, activity impairment, emotional wellbeing and social functioning, as evidenced by the NASH-CHECK data.

Limited previous research has reported worsening symptoms or HRQL in more advanced stages of NASH. A study of patients with NAFLD including patients with NASH found that worsening of fibrosis was independently associated with significantly worse physical HRQL compared with no fibrosis [33]. Another study found that patients with F4 fibrosis reported worse HRQL on several outcome measures compared with patients with F3 fibrosis [10]. The current study supports these findings, and to our knowledge is the first study to report separate utilities and NASH CHECK scores for patients in each fibrosis stage.

There are important limitations in this study which affect our interpretation of the data. Patients with NASH commonly experience comorbidities and people with more severe disease may experience them more frequently. This could lead to an overestimation of the impact of fibrosis on HRQL. In this study, regression analyses estimated the impact of fibrosis stage on EQ-5D utility values after controlling for comorbidities. However, we accept that the research question in this study would have been better addressed with a longitudinal study. Another limitation of the patient survey is that inclusion required a biopsy or fibroscan confirmed fibrosis stage. Although liver biopsy is the gold standard for determining fibrosis stage, it is an invasive procedure that is not performed routinely, particularly in the UK, therefore participants whose fibrosis stage was confirmed through fibroscan were also included in the study. As fibroscan is not the gold standard method for staging fibrosis, this limitation should be considered when interpreting the study results. In addition, although most patients had a biopsy or fibroscan in the 2 years prior to completion of the survey, some participants had not had a biopsy/fibroscan for many years. It is therefore possible that these patients' fibrosis stage may have been misclassified. It was also not possible to recruit large samples of participants with more advanced fibrosis stages, which limits the certainty of the findings. In the DCE, although the attribute selection process aimed to avoid conceptual overlap, it is possible that some participants may have perceived an overlap between some attributes. For example, advancing fibrosis is directly related to a reduction in life expectancy and its possible that participants may have perceived these as correlated. Furthermore, the patient and general population results differed in terms of preference for mode of treatment administration, with patients preferring tablets and the general population preferring injections (stronger preference for more frequent injections). The general public preference for more frequent injections rather than tablets seems counter-intuitive and is unexplained in the current study. There are several possible reasons for this result, one possibility is that although no details about the treatment other than the mode of administration were provided, participants may have inferred that a regular injection may have greater efficacy than a tablet. It is also possible that as the other two attributes were such strong predictors of choice, the significant result for the mode of administration may have been artifactual.

In the general population DCE participants were asked to imagine themselves in the health states described in the choice sets and were provided information to do that. The DCE was kept deliberately simple to aid comprehension. However, it is possible that the general public did not fully understand how the disease would affect daily life. The survey development was informed by a review of existing literature and qualitative research with NASH patients, and the survey content was validated through cognitive debriefing interviews with patients, clinicians and members of the public and through pilot testing. The pilot study also informed various quality control procedures, including the implementation of a minimum time limit for the public survey to exclude any participants speeding through the questionnaire as well as a number of amendments to the survey to improve clarity. Furthermore, as the general population DCE estimated a value for advancing or improving fibrosis stages, a utility estimate was required to ‘anchor’ the values to in order to estimate a value for each stage. In this study the ‘anchor’ was taken from the patient EQ-5D results, using a different anchor would produce different estimates.

## Conclusion

This study uses three different approaches to estimate the disutilities associated with NASH fibrosis stage progression. Each approach showed that disutilities were greater for more advanced fibrosis stages, including in the earlier stages of fibrosis. Larger disutilities were derived from the patient EQ-5D data compared with the general public DCE estimates (which possibly reflects the additional impact of comorbidities). Together these data illustrate the burden of NASH and we believe the importance of gathering the patient perspective on the impact of a disease when developing economic evaluations.

**Summary points**

- Previous utility studies in non-alcoholic steatohepatitis (NASH) have grouped together the earlier fibrosis stages into one health-state utility value.
- Limited research has explored the health-related quality of life impact of NASH at different fibrosis stages.
- Qualitative interviews showed that participants at all fibrosis stages experience symptoms from NASH.
- Fatigue and abdominal pain were common at all stages, patients at more advanced stages reported greater severity.
- Patient-completed EQ-5D-5L and NASH-CHECK also showed greater health-related quality of life impact at more advanced stages.
- Discrete choice experiment (DCE) surveys showed that both patients and the general public showed a strong preference to avoid worse liver fibrosis.
- The study provides estimates of utility values for each fibrosis stage (F1 – F4 decompensated) from three different sources (patient EQ-5D-5L, patient DCE, general public DCE).
- The study provides support for the sensitivity of the EQ-5D-5L in NASH.
