# Supplementary material for: Estimating utility values for non-alcoholic steatohepatitis health states: a discrete choice experiment
Source: J Comp Eff Res. 2024 Jan 16;13(2):e230033. doi: 10.57264/cer-2023-0033 (PMC10842270; doi:10.57264/cer-2023-0033)
Supplement: Supplementary file 1 [file cer-13-230033-s1.docx]

**Appendix file 1: General Public DCE Survey**

## **Exploring preferences for treatments for liver disease: a general public survey**

**Please try to answer all the questions**

Survey introduction

The purpose of this study is to understand people’s views on the impact of a liver disease that can have a very serious impact on people’s health and quality of life. The survey is designed to understand the value that people place on avoiding different stages of the disease and the different treatment aspects.

*It is important that you complete all questions.*

This survey is split into the following three sections:

- In the first section of the survey we will ask you to give us some background information about yourself.
- In the second section we will present you with 15 paired descriptions of hypothetical scenarios and will ask you to choose which option you prefer from each pair.
- In the third section we will present you with another set of 12 paired descriptions of hypothetical scenarios with different symptoms and will ask you to choose which option you prefer from each pair.

## Section 1

In this first part of the survey we would like to ask you some questions about yourself.

1. What is your age?

|_|_| years old

1. What is your gender?
   - Male
   - Female
   - Other
   - Prefer not to answer
2. How would you describe your ethnicity?
   - White (White British/ other)
   - Black (Black British/ Caribbean/ African/ other)
   - Asian (Asian British/ Indian/ Pakistani/ Bangladeshi/ Chinese/ Other)
   - Mixed race
   - Other:
   - Prefer not to answer
3. How would you describe your main activity / employment status?
   - Employed full time
   - Employed part time
   - Full-time homemaker/caregiver
   - Retired
   - Seeking work/ unemployed
   - Unable to work due to disability
   - Student
   - Other __________
   - Prefer not to answer
4. What is the first part of your postcode? _____
5. Do you have a long-term condition that requires you to take medication regularly?
   - Yes
   - No
   - Prefer not to answer
6. Do you suffer from any of the following conditions?

- Cardiovascular disease (heart attack, stroke, angina)
- Type 2 diabetes
- Type 1 diabetes
- High blood pressure
- High cholesterol
- Obesity
- Depression
- Anxiety
- Other, please specify:

1. How close do you live to your nearest hospital?

- Less than 5 miles
- 5-10 miles
- 10-30 miles
- 30 miles or more

## Scenario

We would like to understand the value people place on avoiding different stages of a liver disease and potential aspects of treatment.

We would like you to imagine that you are currently diagnosed with **stage 2** liver disease. At this stage, your liver has some scarring and inflammation, which is caused by a build-up of fat in the liver. As the liver disease gets worse, the scarring on the liver increases and the function declines. There are several stages before a patient experience’s liver failure. As people move through these stages the symptoms they have typically get worse.

Symptoms can include:

- Fatigue or tiredness
- Stomach pain
- Nausea and vomiting
- Bloating
- Itchy skin
- Memory and concentration problems
- Sleep problems

Daily activities, work, home life and social life can all be affected as the disease gets worse.

We would like you to imagine that you are in an early stage of liver disease (**stage 2).**

You will be offered different treatments for your **liver** disease. Treatment options differ in terms of how they are administered (e.g. tablets and injections) and how frequently they are taken.

To understand the importance of these issues, we will present you with different outcomes (in pairs). We ask you to choose which outcome you feel is best.


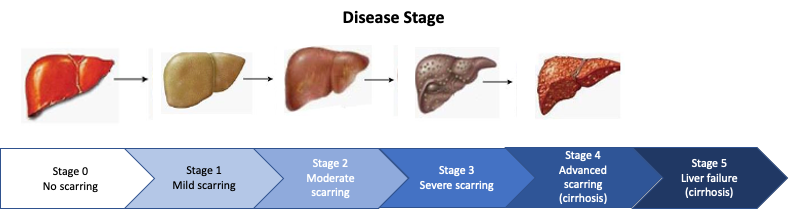


**Q1:** Which of the following statements best describes the scenario that we have asked you to imagine yourself in?

- - 1. You are asked to imagine that you have **stage** **two** liver disease
    2. You are asked to imagine that you have **stage three** liver disease
    3. You are asked to imagine that you have **stage four** liver disease

**Q2:** Which of the stages of the disease is better?

1. You have **stage** **one** liver disease
2. You have **stage** **three** liver disease
3. You have **stage** **five** liver disease

### Length of life

The condition may reduce your overall life expectancy. Depending on the effectiveness of treatment, you can expect:

- Your overall life expectancy to be **reduced by 12 years**

- Your overall life expectancy to be **reduced by 9 years**

- Your overall life expectancy to be **reduced by 6 years**

- Your overall life expectancy to be **reduced by 3 years**

- Your overall life expectancy to be **reduced by 0 years** i.e. not reduced.

Q3. Which of the following is better if you imagine you had **stage 2** liver disease?

- Your overall life expectancy is **reduced by 12 years**
- Your overall life expectancy is **reduced by 0 years** i.e. not reduced.

### Disease stage

If the liver disease is left untreated, the scarring will continue; this leads to cirrhosis, which is advanced, late stage scarring. Treatments aim to slow down and reduce the scarring (disease stage) of your liver. An increase (worsening) in your disease stage can lead to a decline in your liver function and worsening symptoms, including more frequent and more severe fatigue, stomach pain, memory/concentration, nausea/sickness/bloating, itchiness and sleep difficulties. As the disease gets worse it is also likely to impact your ability to carry out daily activities (e.g. work, homelife, leisure) and socialise with friends and family**.**  A reduction in your disease stage (improvement) could improve your liver function, symptoms, ability to carry out daily activities and socialise with friends and family.

Depending on the effectiveness of treatment, in **five years** your disease stage will be at:

- Stage 1 - **mild** liver scarring

- Remain the same/Stage 2 – **moderate** liver scarring

- Stage 3 - **severe** liver scarring

- Stage 4 - **advanced** liver scarring (cirrhosis): you are at higher risk of complications such as loss of liver function and liver cancer.

- Stage 5 - **liver failure:** your liver is losing or has lost most of its function, you may need a liver transplant. You are at higher risk of complications such as liver cancer.

### How the treatment is given

There are different types of treatment available for your liver disease. Depending on the treatment:

- Your treatment is a **tablet** taken **daily** at home

- Your treatment is an **injection** **once a month**

- Your treatment is an **injection every 2 weeks**

**CHOOSING A SCENARIO**

In the questions below you will be presented with a series of **hypothetical** scenarios with two options, Outcome ‘A’ or Outcome ‘B’.

We would like you to imagine you that you are currently diagnosed with **stage 2 liver disease**, meaning you have moderate liver scarring (fibrosis). Your liver function has started to decline, if it declines further (to more severe stages) your symptoms are likely to get worse and this may impact your ability to carry out your daily activities (e.g. e.g. work, homelife, leisure) and socialise.

Some choices may seem contradictory, but we would like you to consider all the information and decide which scenario you would prefer.

**Appendix file 2: Sample DCE choice questions**

**General public fibrosis DCE**

**NASH patient fibrosis DCE**


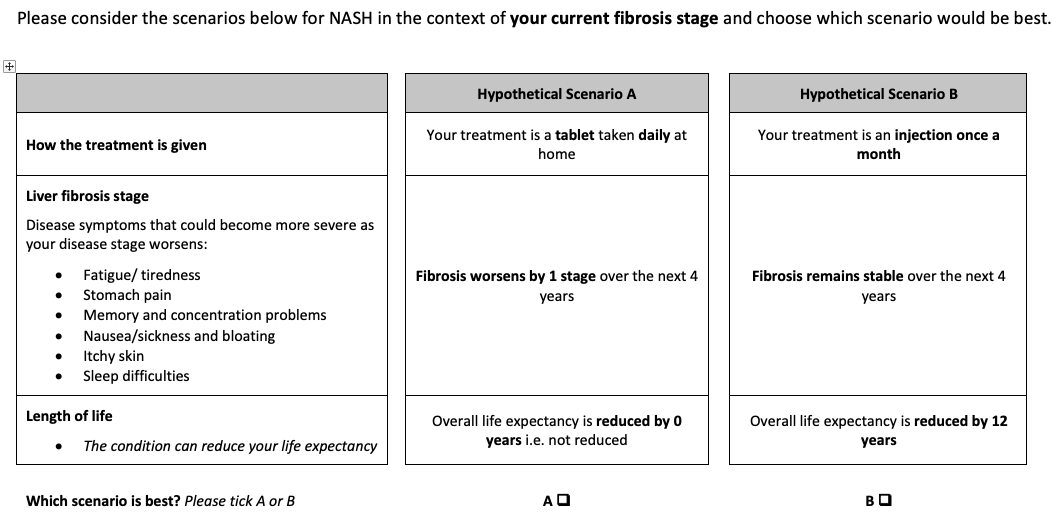


**Appendix File 3. Estimated marginal utilities among NASH patients by fibrosis stage based on results from conditional logit model***

|  | **All (n=154)** | **Stage 1 – 2 (n=77)** | **Stage 3 (n=43)** | **Stage 4 compensated – Stage 4 decompensated (n=34)** |
| --- | --- | --- | --- | --- |
| **Attribute** | **Disutility (95% CI)** | **Disutility (95% CI)** | **Disutility (95% CI)** | **Disutility (95% CI)** |
| **Fibrosis stage** |  |  |  |  |
| Remained the same (ref.) |  |  |  |  |
| Worsened by 1 stage | 0.427 (0.373, 0.481) | 0.259 (0.205, 0.314) | 0.650 (0.508, 0.793) | 0.790 (0.605, 0.976) |
| **How treatment is given** |  |  |  |  |
| Tablet (once daily) (ref.) |  |  |  |  |
| Injection (monthly) | 0.156 (0.092, 0.219) | 0.130 (0.067, 0.194) | 0.095 (-0.080, 0.270) | 0.318 (0.114, 0.522) |
| Injection (biweekly) | 0.387 (0.303, 0.471) | 0.299 (0.213, 0.386) | 0.473 (0.254, 0.692) | 0.614 (0.334, 0.894) |

*Due to small subgroup sample sizes, these analyses were conducted using conditional logit models. Hence, the overall disutility differs to the one reported in the main text, which uses a mixed effects logit model

**Appendix File 4. NASH Check scores and frequency of symptoms by NASH Fibrosis Stage**

| **Characteristic** | | **Stage 1 (n=40)** | **Stage 2 (n=37)** | **Stage 3 (n=43)** | **Stage 4 compensated (n=19)** | **Stage 4 decompensated (n=15)** |
| --- | --- | --- | --- | --- | --- | --- |
|  | | **Mean (SD)** | | | | |
| **NASH Check** |  |  |  |  |  |  |
| Symptom scales |  |  |  |  |  |  |
|  | Abdominal pain | 1.7 (2.1) | 3.3 (2.6) | 5.2 (1.8) | 5.4 (2.3) | 7.3 (1.8) |
|  | Abdominal bloating | 2.3 (2.2) | 4.1 (2.7) | 5.5 (2.4) | 6.8 (2.8) | 7.3 (1.2) |
|  | Physical fatigue | 4.0 (2.4) | 5.8 (2.2) | 6.8 (1.9) | 7.8 (2.3) | 9.0 (1.5) |
|  | Difficulty sleeping | 3.7 (2.5) | 5.1 (2.7) | 6.4 (2.1) | 6.9 (2.5) | 7.8 (2.6) |
|  | Itchy skin | 3.0 (1.8) | 3.6 (2.3) | 4.6 (2.4) | 4.1 (2.8) | 4.9 (1.6) |
|  | Cognitive symptom scale | 1.9 (2.0) | 3.0 (2.3) | 4.2 (1.8) | 4.9 (2.3) | 5.9 (2.1) |
| Activity, emotions, and social scales |  |  |  |  |  |  |
|  | Activity limitations scale | 1.4 (1.9) | 2.7 (2.0) | 4.7 (2.6) | 5.9 (2.4) | 7.5 (1.9) |
|  | Emotional impact scale | 3.8 (2.7) | 4.8 (2.5) | 5.2 (2.7) | 5.9 (2.9) | 7.1 (2.1) |
|  | Social impact scale | 2.5 (2.4) | 3.6 (2.7) | 5.5 (2.5) | 6.1 (2.9) | 8.4 (1.5) |
| **Current symptoms** |  |  |  |  |  |  |
| Fatigue | None of the time, % (n) | 15.0% (6) | 0.0% (0) | 2.3% (1) | 5.3% (1) | 6.7% (1) |
|  | Some of the time, % (n) | 72.5% (29) | 62.2% (23) | 32.6% (14) | 15.8% (3) | 6.7% (1) |
|  | Most of the time, % (n) | 12.5% (5) | 37.8% (14) | 65.1% (28) | 79.0% (15) | 86.7% (13) |
|  |  |  |  |  |  |  |
| Stomach pain | None of the time, % (n) | 62.5% (25) | 27.0% (10) | 4.7% (2) | 10.5% (2) | 0.0% (0) |
|  | Some of the time, % (n) | 35.0% (14) | 56.8% (21) | 79.1% (34) | 36.8% (7) | 20.0% (3) |
|  | Most of the time, % (n) | 2.5% (1) | 16.2% (6) | 16.3% (7) | 52.6% (10) | 80.0% (12) |
|  |  |  |  |  |  |  |
| Memory and concentration problems | None of the time, % (n) | 67.5% (27) | 48.7% (18) | 14.0% (6) | 5.3% (1) | 0.0% (0) |
|  | Some of the time, % (n) | 25.0% (10) | 43.2% (16) | 60.5% (26) | 42.1% (8) | 46.7% (7) |
|  | Most of the time, % (n) | 7.5% (3) | 8.1% (3) | 25.6% (11) | 52.6% (10) | 53.3% (8) |
|  |  |  |  |  |  |  |
| Sleep problems | None of the time, % (n) | 32.5% (13) | 5.4% (2) | 2.3% (1) | 5.3% (1) | 0.0% (0) |
|  | Some of the time, % (n) | 47.5% (19) | 64.9% (24) | 39.5% (17) | 21.1% (4) | 20.0% (3) |
|  | Most of the time, % (n) | 20.0% (8) | 29.7% (11) | 58.1% (25) | 73.7% (14) | 80.0% (12) |

**Appendix File 5. Linear regression results exploring associations between background and clinical characteristics with EQ-5D utility values in the patient sample (n=154)**

|  | **Univariate model** | | **Multivariate model** | |
| --- | --- | --- | --- | --- |
|  | **B coefficient (95% CI)** | **P value** | **B coefficient (95% CI)** | **P value** |
| **Current Fibrosis Stage** |  |  |  |  |
| F1 | REF |  | REF |  |
| F2 | -0.097 (-0.209, 0.015) | 0.090 | -0.083 (-0.188, 0.022) | 0.119 |
| F3 | -0.255 (-0.363, -0.147) | <0.001 | -0.244 (-0.360,-0.129) | <0.001 |
| F4a | -0.516 (-0.653, -0.379) | <0.001 | -0.449 (-0.586,-0.313) | <0.001 |
| F4b | -0.646 (-0.795, -0.498) | <0.001 | -0.585 (-0.738,-0.432) | <0.001 |
| **Age** | -0.009 (-0.013, -0.005) | <0.001 | 0.000 (-0.004, 0.004) | 0.962 |
| **Gender** |  |  | – | – |
| Male | REF |  |  |  |
| Female | 0.044 (-0.060, 0.149) | 0.405 |  |  |
| **Years diagnosed** |  |  |  |  |
| ≤2 years | REF |  | – | – |
| 2-4 years | -0.022 (-0.147, 0.104) | 0.733 | – | – |
| 4-6 years | -0.124 (-0.269, 0.021) | 0.093 | – | – |
| >6 years | -0.347 (-0.467, -0.226) | <0.001 | – | – |
| **Employment status** |  |  |  |  |
| Employed full time | REF |  | – | – |
| Employed part time | 0.028 (-0.156, 0.212) | 0.766 | – | – |
| Homemaker/caregiver | -0.013 (-0.169, 0.144) | 0.874 | – | – |
| Retired | -0.304 (-0.425, -0.182) | <0.001 | – | – |
| Unemployed | -0.118 (-0.340, 0.103) | 0.293 | – | – |
| Unable to work/disability | -0.437 (-0.569, -0.305) | <0.001 | – | – |
| Other | 0.014 (-0.170, 0.198) | 0.881 | – | – |
| **Comorbidites** |  |  |  |  |
| Cardiovascular disease | -0.286 (-0.437, -0.135) | <0.001 | -0.164 (-0.278,-0.049) | 0.005 |
| Type 2 diabetes | -0.196 (-0.302, -0.090) | <0.001 | -0.029 (-0.114, 0.056) | 0.503 |
| Hypertension | -0.104 (-0.212, 0.003) | 0.058 | – | – |
| High cholestorol | -0.146 (-0.254, -0.037) | 0.009 | -0.057 (-0.140, 0.026) | 0.176 |
| Obesity | -0.197 (-0.328, -0.066 | 0.003 | -0.076 (-0.173, 0.020) | 0.120 |
| Depression | -0.152 (-0.285, -0.020) | 0.024 | -0.134 (-0.231, -0.036) | 0.008 |
| Anxiety | -0.208 (-0.358, -0.057) | 0.007 | -0.212 (-0.325, -0.099) | <0.001 |
| Other condition | -0.135 (-0.309, 0.039) | 0.126 | – | – |
| **Model statistics** |  | |  | |
| *Constant* | – | | *0.867* | |
| *R-squared* | – | | *0.561* | |
| *RMSE* | – | | *0.224* | |
